# The *Scutellaria baicalensis* R2R3-MYB Transcription Factors Modulates Flavonoid Biosynthesis by Regulating GA Metabolism in Transgenic Tobacco Plants

**DOI:** 10.1371/journal.pone.0077275

**Published:** 2013-10-15

**Authors:** Yuan Yuan, Chong Wu, Yunjun Liu, Jian Yang, Luqi Huang

**Affiliations:** 1 National Resource Center for Chinese Materia Medica, Academy of Chinese Medical Sciences, Beijing, China; 2 Institute of Crop Science, Chinese Academy of Agricultural Sciences, Beijing, China; Key Laboratory of Horticultural Plant Biology (MOE), China

## Abstract

R2R3-MYB proteins play role in plant development, response to biotic and abiotic stress, and regulation of primary and secondary metabolism. Little is known about the R2R3-MYB proteins in *Scutellaria baicalensis* which is an important Chinese medical plant. In this paper, nineteen putative *SbMYB* genes were identified from a *S. baicalensis* cDNA library, and eleven R2R3-MYBs were clustered into 5 subgroups according to phylogenetic reconstruction. In the *S. baicalensis* leaves which were sprayed with GA_3_, *SbMYB2* and *SbMYB7* had similar expression pattern with *SbPALs*, indicating that SbMYB2 and SbMYB7 might be involved in the flavonoid metabolism. Transactivation assay results showed that SbMYB2 and SbMYB7 can function as transcriptional activator. The expression of several flavonoid biosynthesis-related genes were induced or suppressed by overexpression of *SbMYB2* or *SbMYB7* in transgenic tobacco plants. Consistent with the change of the expression of *NtDH29 and NtCHI*, the contents of dicaffeoylspermidine and quercetin-3,7-O-diglucoside in *SbMYB2*-overexpressing or *SbMYB7*-overexpressing transgenic tobacco plants were decreased. The transcriptional level of *NtUFGT* in transgenic tobacco overexpressing *SbMYB7* and the transcriptional level of *NtHCT* in *SbMYB2-*overexpressing tobacco plants were increased; however the application of GA_3_ inhibited the transcriptional level of these two genes. These results suggest that SbMYB2 and SbMYB7 might regulate the flavonoid biosynthesis through GA metabolism.

## Introduction

MYB proteins present in all eukaryotes and play roles in a variety of plant-specific processes, as evidenced by their extensive functional characterization in *Arabidopsis* (*Arabidopsis thaliana*) [1], maize (*Zea mays*) [2], rice (*Oryza sativa*) [3], petunia (*Petunia hybrida*) [4], grapevine (*Vitis vinifera* L.) [5], poplar (*Populus tremuloides*) [6] and apple (*Malus domestica*) [7]. The increasing availability of plant genome sequence information has allowed comparisons and a better understanding of the evolution of this large family of transcription factors. 

Most plant MYB proteins belong to the R2R3-MYB subfamily [8], and the *Arabidopsis* R2R3-type MYB factors encoded by the *AtMYB* genes have been categorized into 22 subgroups on the basis of the conserved amino acid sequence motifs [9]. *Arabidopsis* R2R3-MYB proteins have been found to be involved in primary and secondary metabolism, cell fate and identity, developmental processes and responses to biotic and abiotic stresses [10]. Some R2R3-MYB proteins are also involved in the regulation of the flavonoid biosynthetic pathway [11]. Overexpression of *AtMYB75/PAP1* and *AtMYB90/PAP2* resulted in a massive activation of phenylpropanoid biosynthetic genes and enhanced the accumulation of lignin, hydroxycinnamic acid esters, and purple anthocyanins [12]. AtMYB4 was shown to negatively regulate the expression of cinnamate 4-hydroxylase gene, then repress the synthesis of sinapoyl malate. 

**Figure 1:**
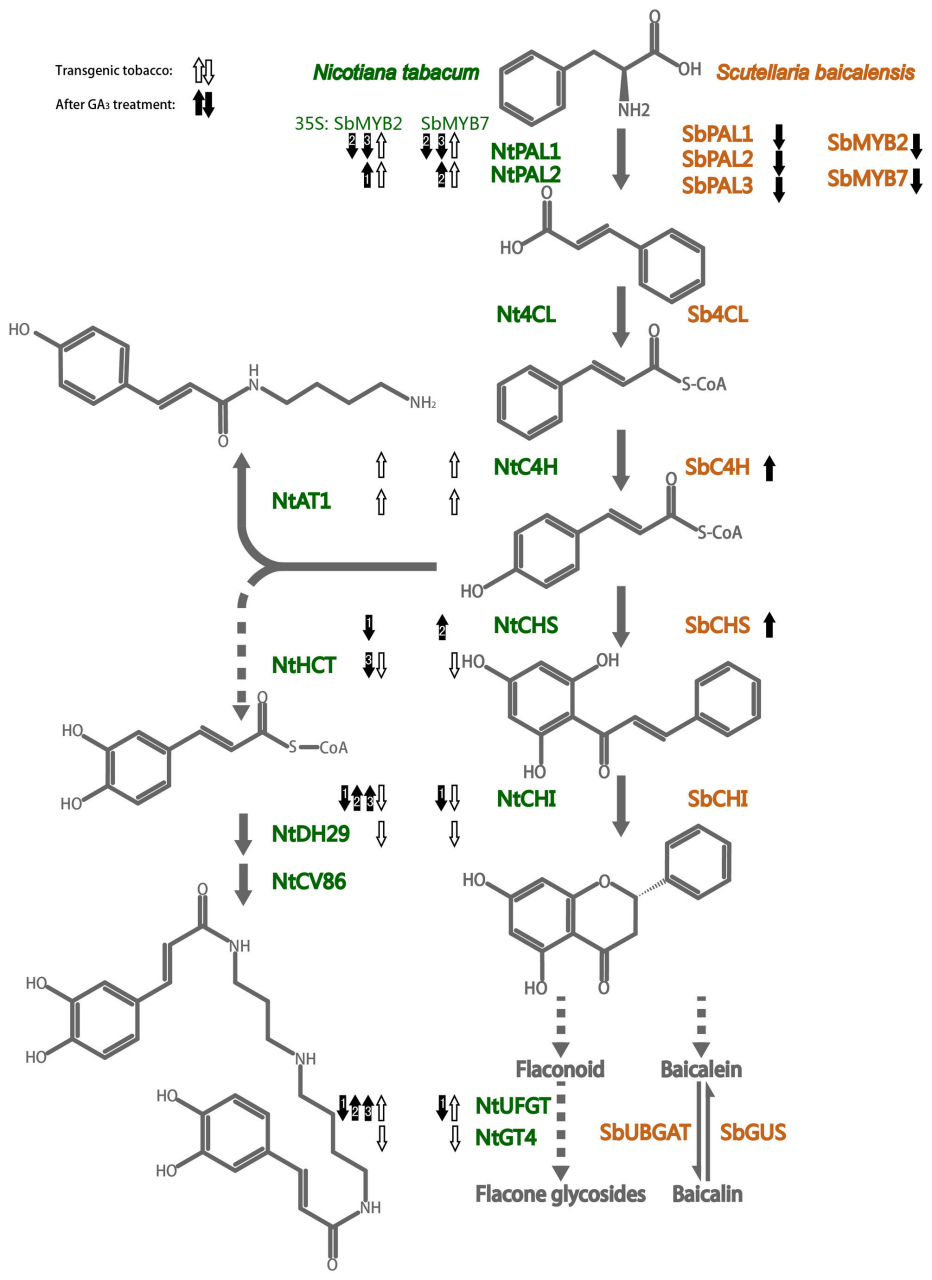
Phenylpropanoid and flavonoid biosynthesis in *S. baicalensis* and tobacco.

The roots of *Scutellaria baicalensis* Georgi are used to treat various diseases in Chinese traditional medicine. The active compounds of *S. baicalensis* include baicalin, baicalein, wogonoside, wogonin, neobaicalein, visidulin I, and oroxylin A, and these compounds exhibit anti-inflammatory, anti-tumor, and anti-HIV activities [13]. Baicalin is synthesized via the phenylpropanoid pathway by the activities of several enzymes (Figure 1), including phenylalanine ammonia-lyase (PAL), cinnamate 4-hydroxylase (C4H), 4-coumarate:CoA ligase (4CL), chalcone synthase (CHS) and chalcone isomerase (CHI) [14]. β-glucuronidase (GUS) catalyze baicalin to baicalein [15,16]. Baicalein can be catalyzed back to baicalin by UDP-glucuronate: baicalein 7-O-glucuronosyltransferase (UBGAT) [17]. In tobacco, coumaroyl- phenylpropanoids were firstly synthesized by PAL, 4CL and C4H, and formed to caffeoyl- and feruloyl- phenylpropanoids by p-coumarate 3-hydroxylase and caffeic acid 3-O-methyltransferase [18]. CHS and CHI were also important genes in biosynthesis of anthocyanidins pathway [19]. Hydroxycinnamoyl-coenzyme A: putrescine acyltransferase (AT1) responsible for caffeoylputrescine biosynthesis in tobacco, and another acyltransferase DH29 was specific for spermidine conjugation to mediate the initial acylation step in dicaffeoylspermidine formation [20]. Hydroxycinnamoyl transferase (HCT) and caffeoyl-CoA O-methyltransferase (CCoAOMT) catalyzed the synthesis of shikimate and quinate esters in phenylpropanoid biosynthesis [21]. And both glucosyltransferase (GT) and UDP-glucose:flavonoid 3-O-glucosyltransferase (UFGT) were flavonoid-glucosyltransferases in tobacco (Figure 1) [22]. 

In our previous work, proteomics analysis showed that the protein level of a putative R2R3-MYB transcription factor in *S. baicalensis* roots was increased under water deficit condition. This R2R3-MYB has high identity with AtMYB113 which is involved in the regulation of anthocyanin biosynthesis, indicating that the R2R3-MYB transcription factor is also involved in flavonoid biosynthesis in *S. baicalensis*. The expression levels of several proteins related to GA metabolism were also affected. These prompted us to consider the possible linkage between flavonoid accumulation and hormone metabolism [23]. 

In this paper, we obtained nineteen *S. baicalensis* MYB transcription factors from a cDNA library, and performed a phylogeny and expression patterns analysis to yield an overview of the R2R3-MYB gene family in *S. baicalensis*. We further confirmed that SbMYB2 and SbMYB7 play roles in flavonoids biosynthesis.

## Results

### Identification of *R2R3-MYB* genes from *S*. *baicalensis*

We have developed a *S. baicalensis* full-length cDNA library (unpublished work). To identify R2R3 type MYB genes in *S. baicalensis*, a preliminary BLASTX search was performed using NR database in full-length cDNA library. Only hits with *E* values below e^-30^ were considered as members of this gene family. Eleven SbMYB genes have R2R3-MYB conserved domains and motifs, and their deduced proteins showed different lengths, isoelectric points, and molecular weights (Table S1; Table S2). The sequences of these nineteen *SbMYB* genes have been submitted to the GenBank with the accession number KC990835, KC990836, KF008651-KF008667.

**Figure 2:**
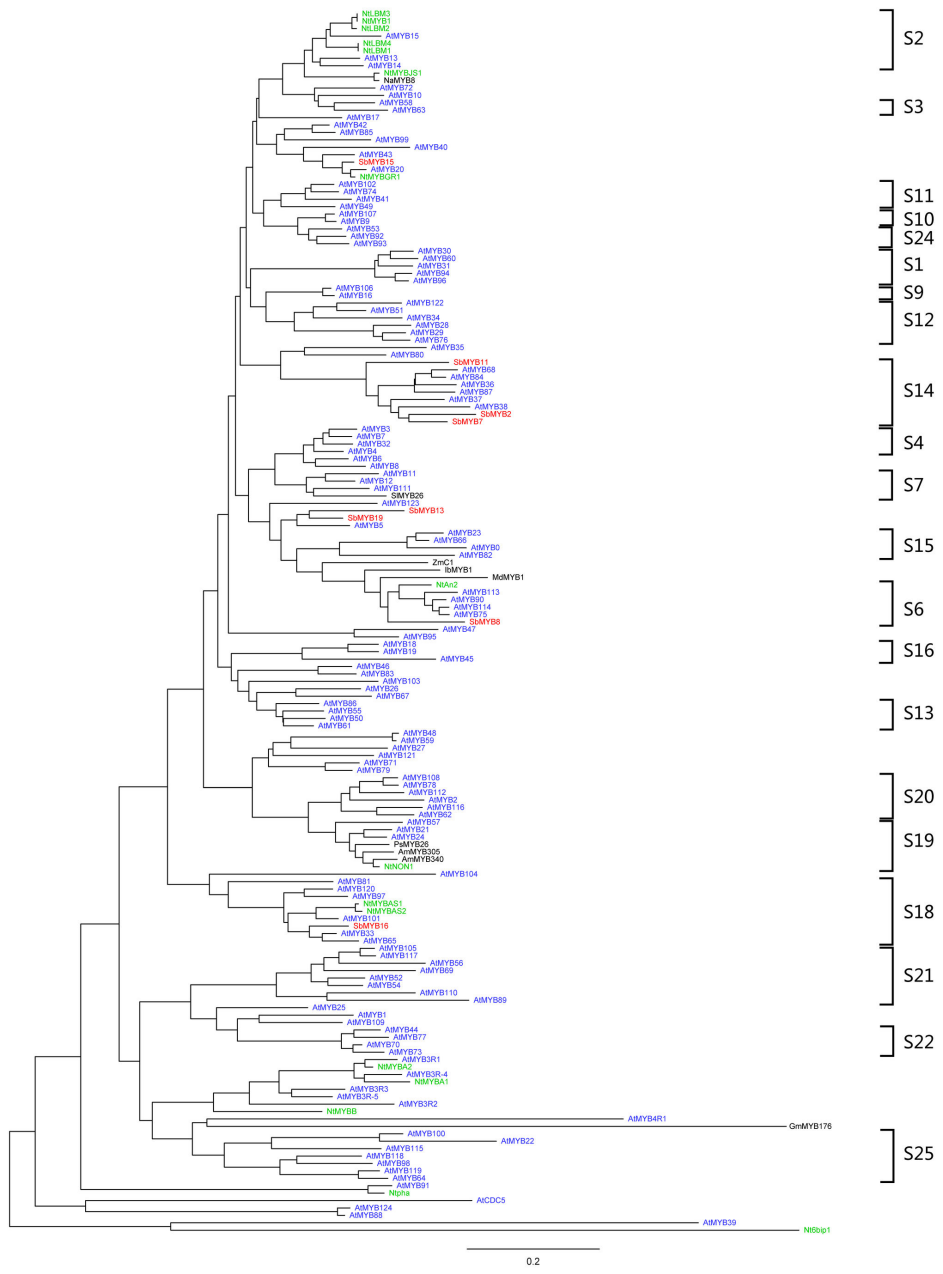
Neighbor-joining tree representing relationships among MYB proteins from *Scutellaria baicalensis*, *Arabidopsis* and Nicotiana. The proteins are clustered into 23 subgroups, which are designated with a subgroup number (e.g., S1).

Based on sequence similarity, the identified *S. baicalensis* R2R3-MYB proteins were clustered into 5 subgroups, according to clades with at least 50% bootstrap support (Figure 2). During the subfamily classification of the MYB genes, we also took into account the results of Stracke et al. [8] and Dubos et al. [26] for AtMYBs. The validity of our phylogenetic reconstruction is confirmed by the fact that it shows the same subgroups as those observed in previously constructed phylogenetic trees. SbMYB2, SbMYB7 and SbMYB11 belong to subgroup S14. SbMYB13 and SbMYB19 were clustered with OsMYB4 and ATMYB5, and SbMYB15 was clustered with AtMYB20, AtMYB43, AtMYB85, AtMYB42, AtMYB40 and AtMYB99. Only SbMYB8 belongs to subgroup S6, and SbMYB16 belongs to subgroup S18. In general, the gene functions of a clade appear highly but not absolutely conserved across plant species. Thus, knowledge of the gene functions of certain members will facilitate confirmation of paralogous and orthologous relationships. 

### The expression pattern of *S*. *baicalensis R2R3-MYB* genes and flavonoid biosynthesis-related genes

**Figure 3:**
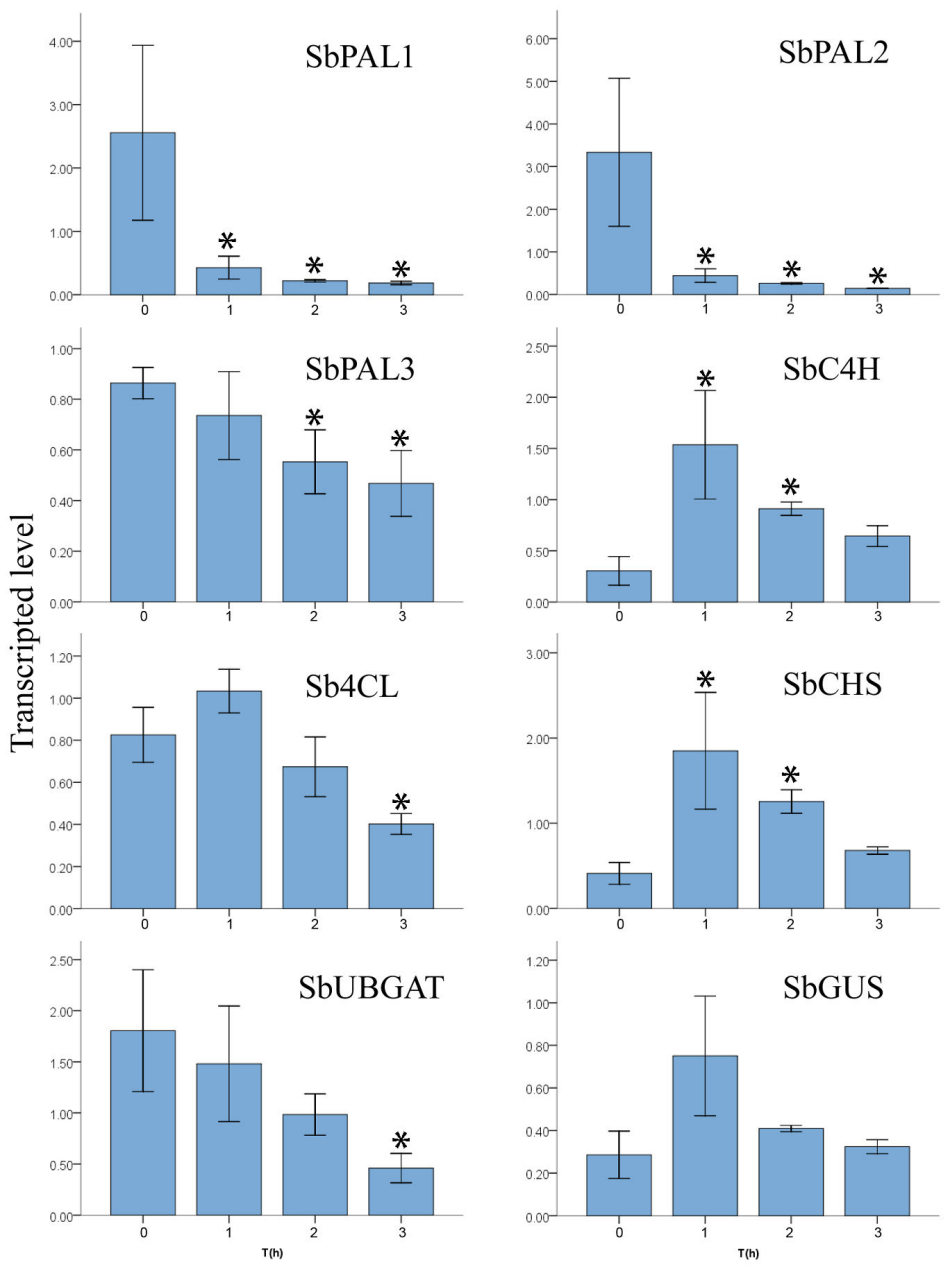
Effects of GA_3_ treatment on the expression of flavonoid biosynthesis related genes in *S. baicalensis*. RT-PCR analysis of expression of *SbPAL1*, SbPAL2, *SbPAL3*, *SbC4H*, *Sb4CL*, *SbCHS*, *SbGUS*, and *SbUBGAT* in leaves of *S. baicalensis* after spraying GA_3_. Vertical bars indicate the standard deviation of three biological replicates. Asterisks indicate a significant difference at the *P*<0.05 level.

**Figure 4:**
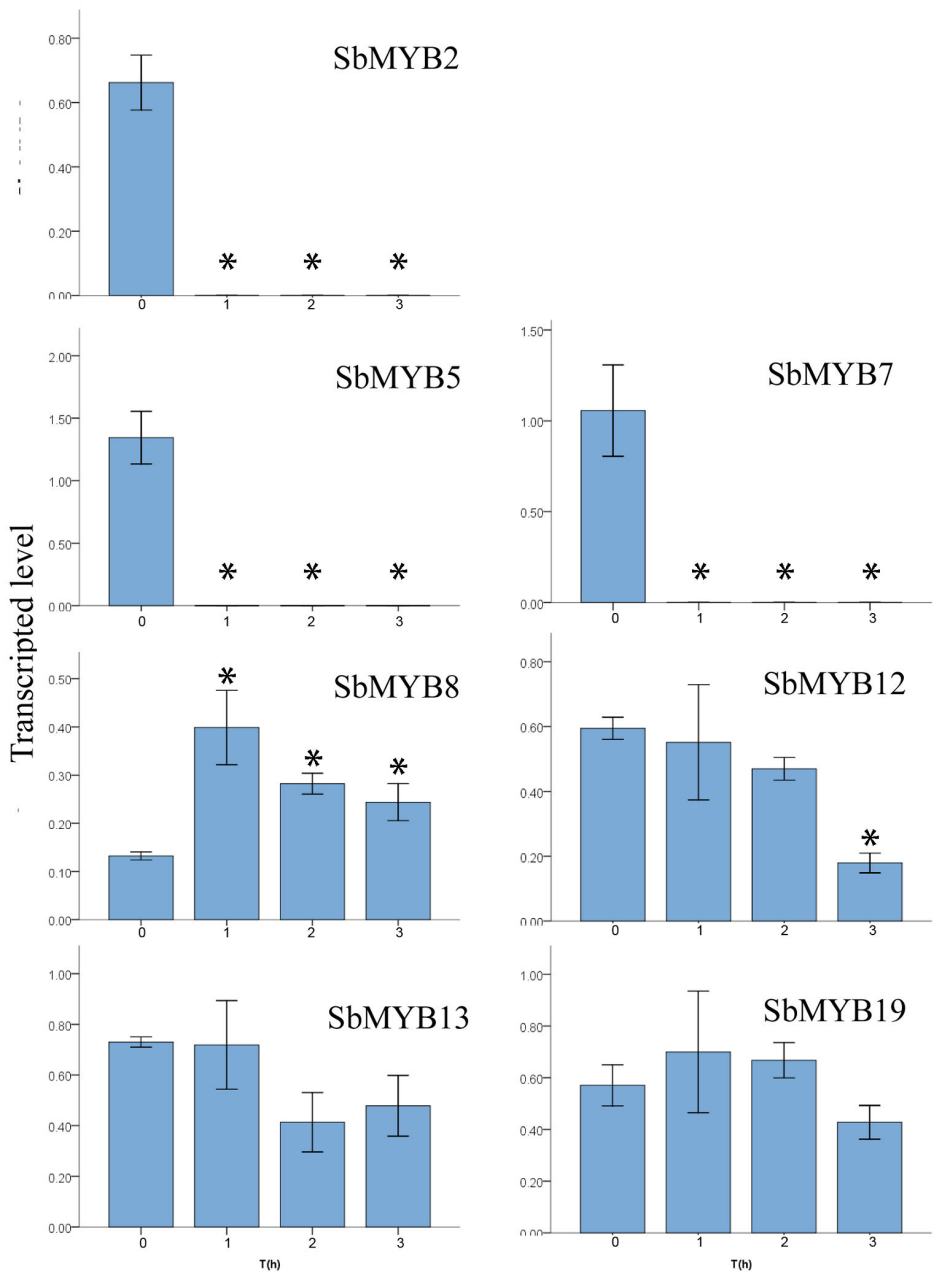
Effects of GA_3_ treatment on the expression of MYB genes in *S. baicalensis*. RT-PCR analysis of transcriptional level of *SbMYB2*, *SbMYB5*, *SbMYB7*, *SbMYB8*, *SbMYB12*, *SbMYB13*, and *SbMYB19* in leaves of *S. baicalensis* at 0, 1, 2, 3 h after spraying GA_3_. Vertical bars indicate the standard deviation of three biological replicates. Asterisks indicate a significant difference at the *P*<0.05 level.

The flavonoid accumulation in *S. baicalensis* might be related with GA hormone metabolism and some R2R3-MYB proteins might be involved in the flavonoid accumulation [23]. The expression of some *R2R3-MYB* genes and the flavonoid biosynthesis-related genes were investigated in the *S. baicalensis* leaves which were sprayed with GA_3_. The results showed that exogenous GA_3_ decreased the expression of *Sb4CL*, *SbUBGAT*, *SbPAL1*, *SbPAL2* and *SbPAL3*, whereas the expression of *SbC4H* and *SbCHS* were increased by GA_3_ treatment (Figure 3). The expression of *SbMYB2*, *SbMYB5*, *SbMYB7* and *SbMYB12* was decreased after GA_3_ treatment, however GA_3_ treatment increased the expression of *SbMYB8* (Figure 4). *SbMYB2* and *SbMYB7* had similar expression pattern with *SbPALs*, indicating that SbMYB2 and SbMYB7 might be involved in the flavonoid metabolism. The functions of these two genes were further investigated.

### Subcellular localization of SbMYB2 and SbMYB7

**Figure 5:**
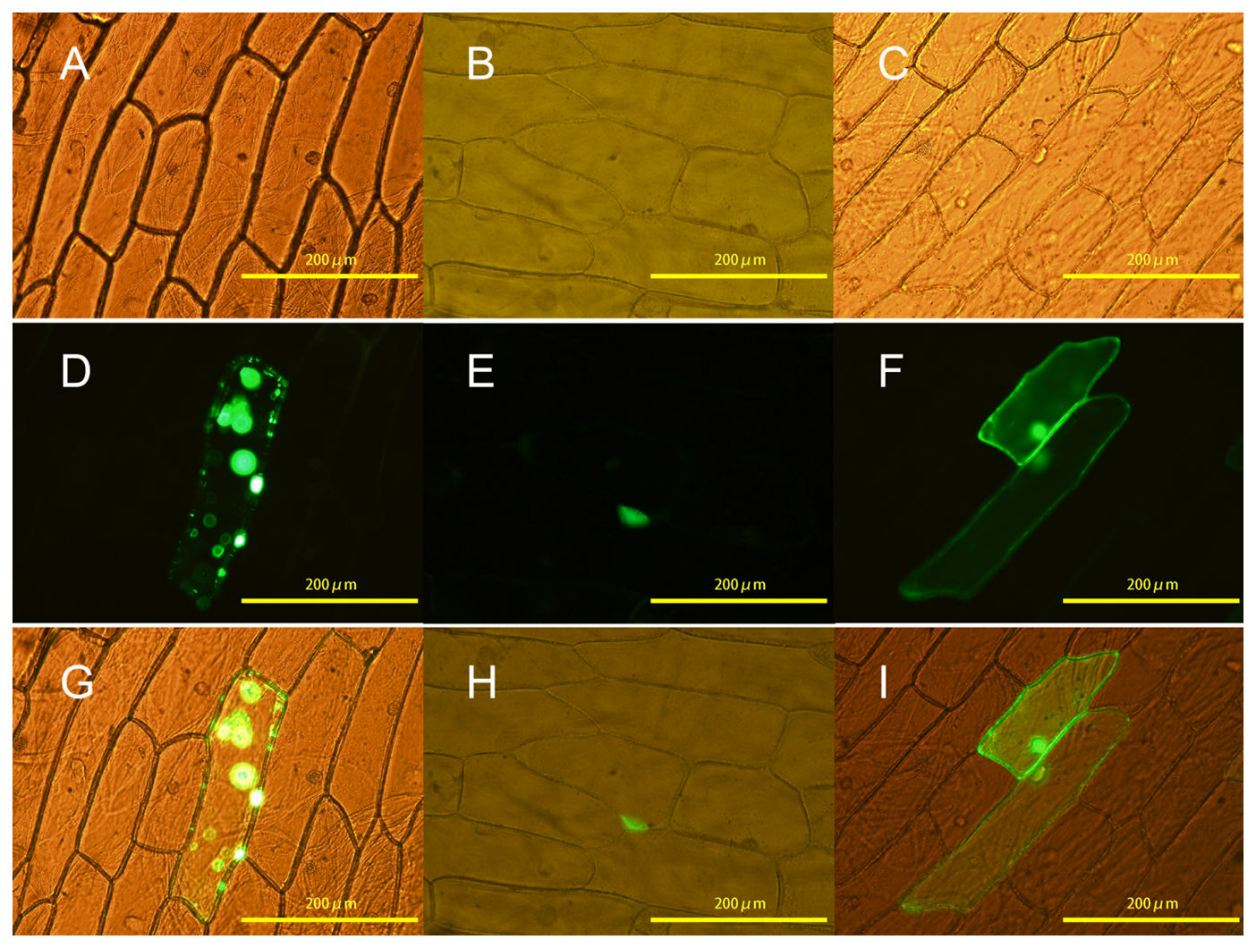
Subcellular localization of SbMYB2 and SbMYB7. The recombinant constructs of the *SbMYB2-GFP* and *SbMYB7-GFP* fusion gene and *GFP* alone were transformed into onion (*Allium cepa*) epidermal cells by particle bombardment. A, D, G, pGEM-SbMYB2; B, E, H, pGEM-SbMYB7; C, F, I, empty vector pE3025.

Firstly, we determine the subcellular localization of SbMYB2 and SbMYB7. The full-length cDNA sequence of *SbMYB2* and *SbMYB7* were fused in front of the 5’ terminus of *GFP* reporter gene under the control of CaMV 35S promoter with the correct reading frame, respectively. The recombinant constructs of the *SbMYB2-GFP* and *SbMYB7-GFP* fusion gene and *GFP* alone were transformed into onion (*Allium cepa*) epidermal cells by particle bombardment. SbMYB7-GFP fusion protein accumulated mainly in the nucleus, suggesting that SbMYB7 is a nucleus-localized protein. Whereas SbMYB2-GFP fusion protein is located not only in nucleus but also in some other plastids，and GFP alone was present throughout the whole cell (Figure 5). These results are consistent with the predicted localization results (Table S3). 

### Transactivation assay of SbMYB2 and SbMYB7

**Figure 6:**
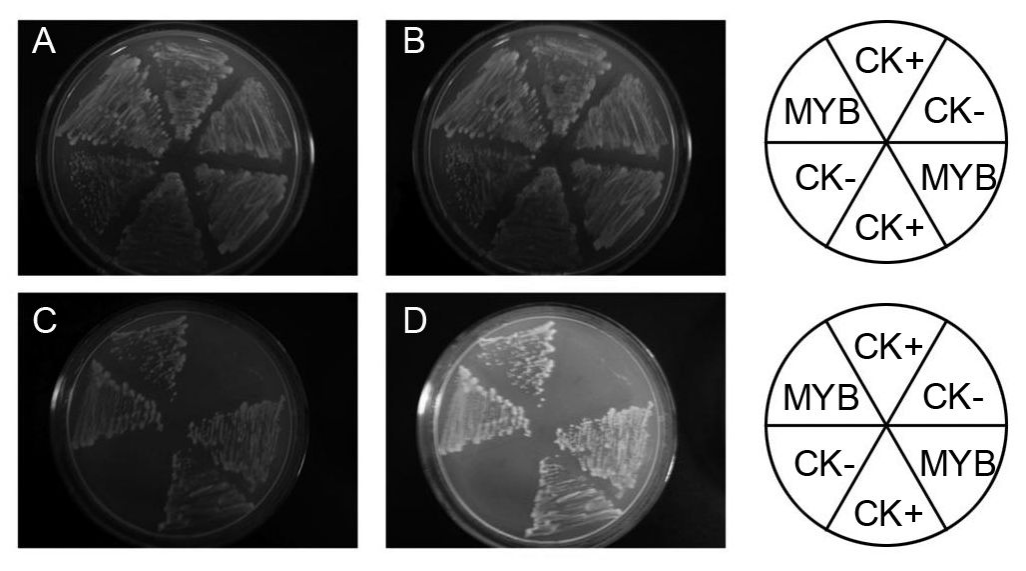
Transactivation assay of SbMYB2 and SbMYB7. A,C, pBD-SbMYB2; B,D, pBD-SbMYB7. pGAL4 and pBD-GAL4 was used as a positive control and negative control, respectively.

A yeast GAL4 system was used to determine the transcription activity of SbMYB2 and SbMYB7. The full-length cDNA of *SbMYB2* and *SbMYB7* was fused to the GAL4 DNA-binding domain of the pGBKT7 vector, and the fusion plasmid pBD-SbMYB2 and pBD-SbMYB7 was transformed into the yeast strain YGR2. Figure 6 showed that yeasts transformed with pBD-SbMYB2 or pBD-SbMYB7 could grow on the selection synthetic dextrose mediums lacking tryptophan and adenine (SD/-Trp/-Ade), and on the medium lacking tryptophan, adenine, and histidine (SD/-Trp/-Ade/-His). A healthy growth of yeast on both media were detected in the transformants containing the full-length cDNA of *SbMYB2* and *SbMYB7* compared with the control yeast transformed with empty vector, These results suggests that the SbMYB2 and SbMYB7 protein can function as transcriptional activator.

### Molecular characterization of transgenic tobacco lines overexpressing *SbMYB2* and *SbMYB7*

To investigate the function of SbMYBs in plants, *SbMYB2* and *SbMYB7* were transformed into tobacco plants, respectively. The integration of *SbMYB2* and *SbMYB7* was confirmed using PCR analysis (Figure S1). The real-time RT-PCR analysis results showed that the expression of *SbMYB2* and *SbMYB7* was markedly increased in the transgenic plants (Table S4). Three independent transgenic lines (e10-25, e10-26 and e10-29) overexpressing *SbMYB2* and three independent transgenic lines (e18-53, e18-b and e18-d) overexpressing *SbMYB7* were selected for further analysis.

### SbMYB2 and SbMYB7 regulates the expression of flavonoid biosynthesis-related genes

**Figure 7:**
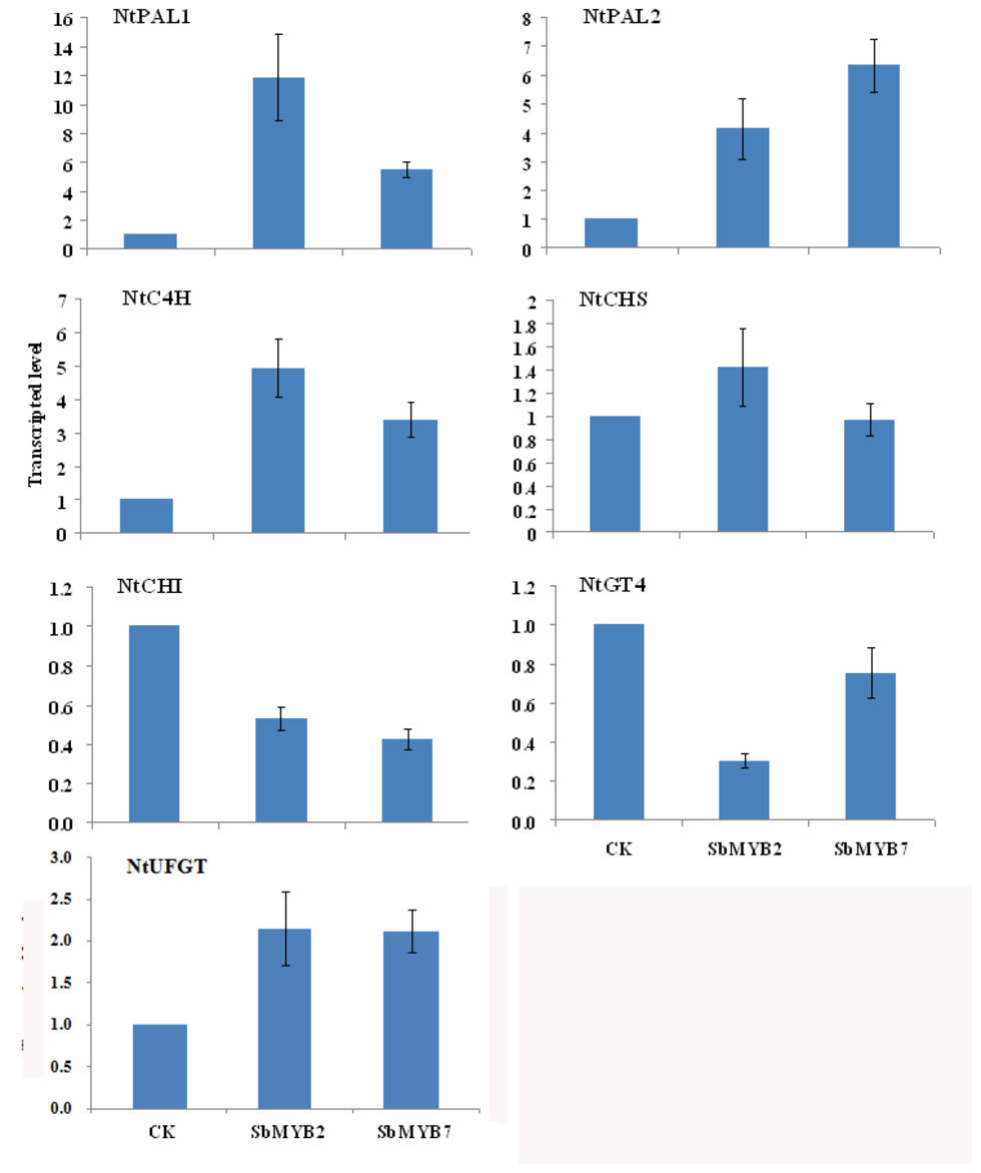
Transcriptional level of flavonoid biosynthesis related genes in T1 transgenic tobacco plants. Quantitative real-time PCR analysis of transcription level of *NtPAL1*, *NtPAL2*, *NtC4H*, *NtCHS*, *NtCHI*, *NtGT4* and *NtUFGT* in leaves of *SbMYB2*-overexpressing and *SbMYB7*-overexpressing tobacco plants.

**Figure 8:**
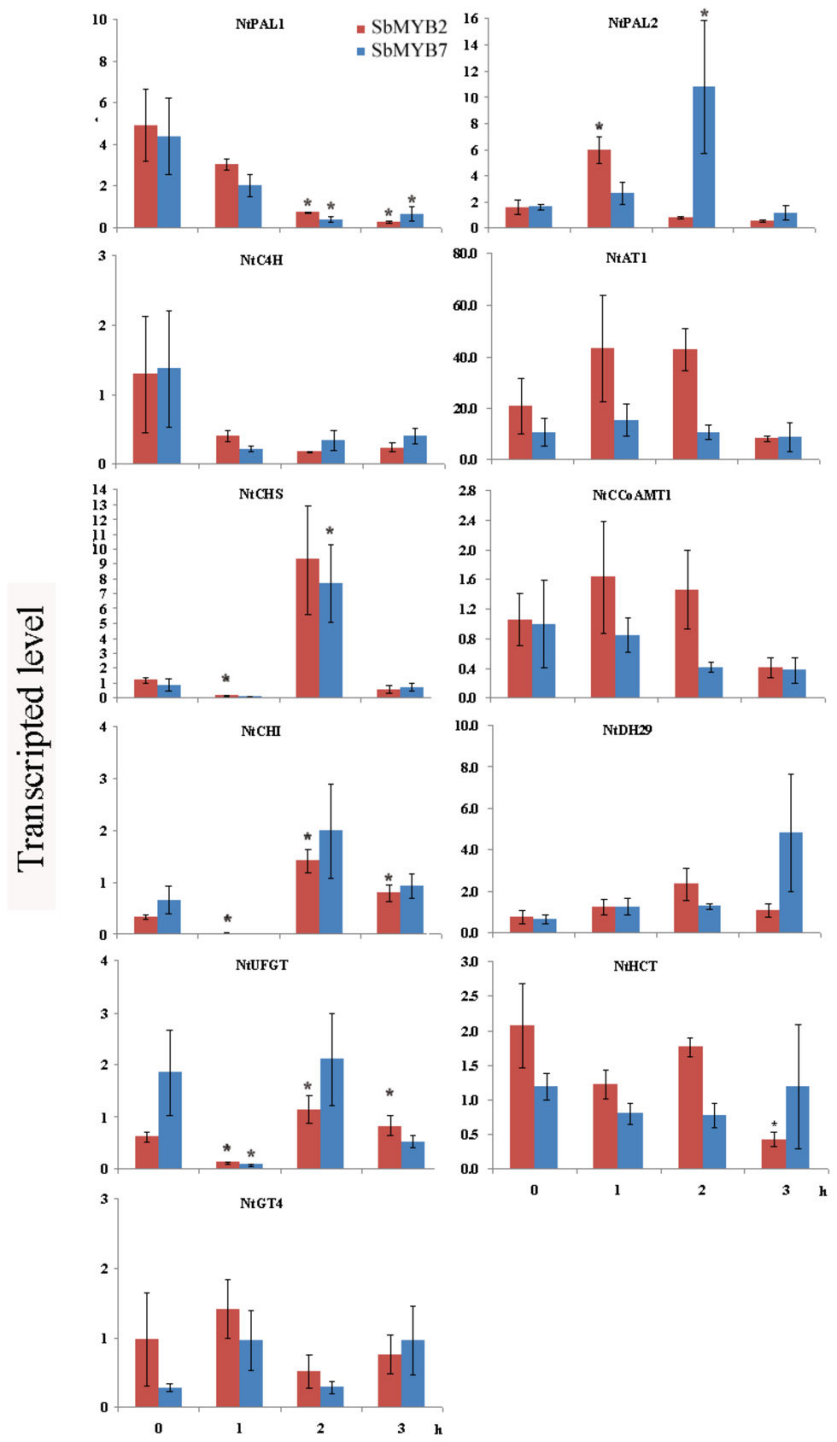
Transcriptional level of flavonoid biosynthesis related genes in T1 transgenic tobacco after GA_3_ treatment. Quantitative real-time PCR analysis of transcript level of *NtPAL1*, *NtPAL2*, *NtC4H*, *NtCHS*, *NtCHI*, *NtGT4*, *NtUFGT*, *NtAT1*, *NtCCoAMT1*, *NtDH29* and *NtHCT* in leaves of *SbMYB2*-overexpressing and *SbMYB7*-overexpressing tobacco plants at 0, 1, 2, 3 h after spraying GA_3_, respectively. Vertical bars indicate the standard deviation of three biological replicates. Asterisks indicate a significant difference at the *P*<0.05 level.

To investigate whether SbMYB2 and SbMYB7 regulates the flavonoid biosynthesis, the expression of several flavonoid biosynthesis-related genes including *NtPAL1*, *NtPAL2*, *NtC4H*, *NtCHS*, *NtCHI*, *NtUFGT*, *NtGT4*, *NtAT1*, *NtDH29*, *NtHCT* and *NtCCoAMT1* in transgenic tobacco plants were measured using real-time RT-PCR with the specific primers. The expression of *NtPAL1*, *NtPAL2*, *NtC4H* and *NtUFGT* were markedly induced in transgenic tobacco plants expressing *SbMYB2* or *SbMYB7*, indicating that SbMYB2 or SbMYB7 positively regulates the expression of these genes. The expression of *NtCHI* was decreased in transgenic plants overexpressing *SbMYB2* or *SbMYB7* and overexpression of *SbMYB2* decreased the expression of *NtGT4* (Figure 7), indicating that SbMYB2 negatively regulate the expression of *CHI* and *GT4*. The expression of *NtAT1* was increased and the transcriptional level of *NtDH29* was slightly decreased in both *SbMYB2*-overexpressing and *SbMYB7*-overexpressing transgenic tobacco plants (Figure 8). Because *NtDH29* is an important gene which is involved in the biosynthesis of dicaffeoylspermidine [20], above results indicates that *SbMYB2* and *SbMYB7* could be related to the dicaffeoylspermidine formation. The expression of flavonoid related genes in wild type tobacco plants were not affected by GA treatment (Table S5). 

### SbMYB2 and SbMYB7 negatively regulated the synthesis of dicaffeoylspermidine and flavone in transgenic tobacco

**Figure 9:**
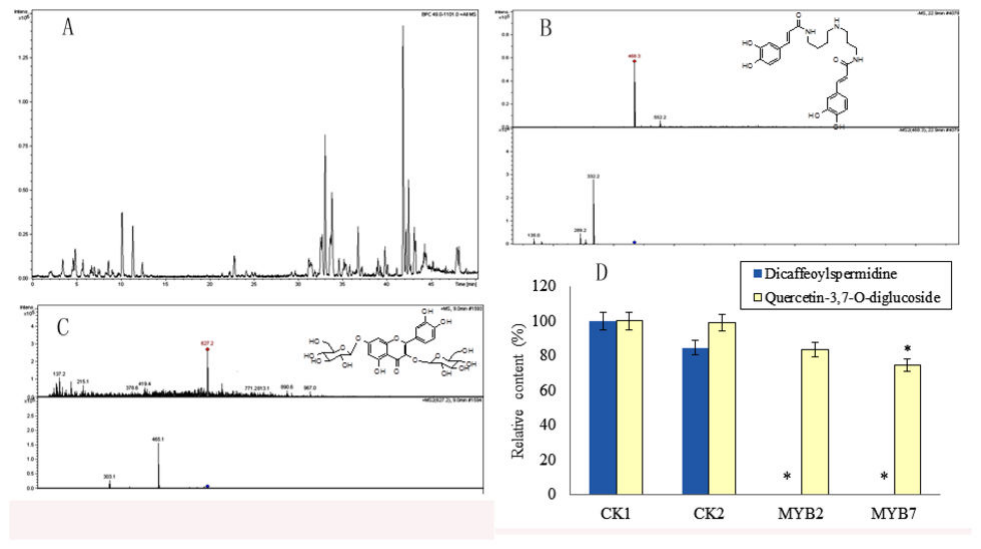
Chemical analysis of *SbMYB2*-overexpressing and *SbMYB7*-overexpressing transgenic tobacco plants. A, UPLC analysis. B,C Dicaffeoylspermidine and quercetin-3,7-O-diglucoside were identified using LC-MS and LC-MS/MS. D, content of dicaffeoylspermidine and quercetin-3,7-O-diglucoside in transgenic tobacco.

The overexpression of *SbMYB2* or *SbMYB7* led to the change of the expression of flavonoid biosynthesis-related genes, indicating that SbMYB2 or SbMYB7 might regulate the accumulation of flavonoid. To investigate whether the overexpression of *SbMYB2* or *SbSMB7* in transgenic tobacco plants affected the accumulation of flavonoid, we performed HPLC analysis of the leaf samples. Two peaks having significant areas were inhibited in both transgenic plants overexpressing *SbMYB2* or *SbMYB7*, compared with wild type plants, and were identified as dicaffeoylspermidine and quercetin-3,7-O-diglucoside by LC-MS analysis (Figure 9). Overexpression of *SbMYB2* or *SbMYB7* decreased the accumulation of dicaffeoylspermidine and quercetin-3,7-O-diglucoside (Figure 9D), indicating that SbMYB2 and SbMYB7 negatively regulated flavonoid synthesis in transgenic tobacco plants.

### Exogenous GA_3_ affected the expression of flavonoid pathway genes in transgenic tobacco plants over-expressing *SbMYB2* or *SbMYB7*

To further analyze the function of SbMYB2 and SbMYB7 in possible linkage among flavonoid accumulation and GA metabolism, exogenous GA_3_ were sprayed on the leaves of transgenic plants overexpressing *SbMYB2* or *SbMYB7*, and the expression of several flavonoid pathway genes were measured by real-time RT-PCR. Expression of *NtPAL1* was decreased in both *SbMYB2*-overexpressing and *SbMYB7*-overexpressing transgenic plants at 2 and 3h after spay exogenous GA_3_. The expression of *NtPAL2* was markedly increased in *SbMYB2*-overexpressing transgenic plants at 1h after spaying exogenous GA_3_, whereas was increased in *SbMYB7*-overexpressing transgenic plants at 2h after exogenous GA_3_ treatment. Transcriptional level of *NtCHS* was increased in *SbMYB2* and *SbMYB7*-overexpressing transgenic plants at 3h after spaying exogenous GA_3_. The transcriptional levels of *NtCHI* and *NtUFGT* were decreased at 1h and increased at 2 and 3h in *SbMYB2*-overexpressing transgenic plants after spaying exogenous GA_3_, and decreased at 1h in *SbMYB7*-overexpressing transgenic plants after spaying exogenous GA_3_. The expression of *NtHCT* was decreased in *SbMYB2*-overexpressing transgenic plants at 3h after spaying exogenous GA_3_ (Figure 8). Gene expression pattern without GA application was also investigated and no difference was observed (Table S6). 

### SbMYBs bind with the box L sequence of the *PAL* promoter

**Figure 10:**
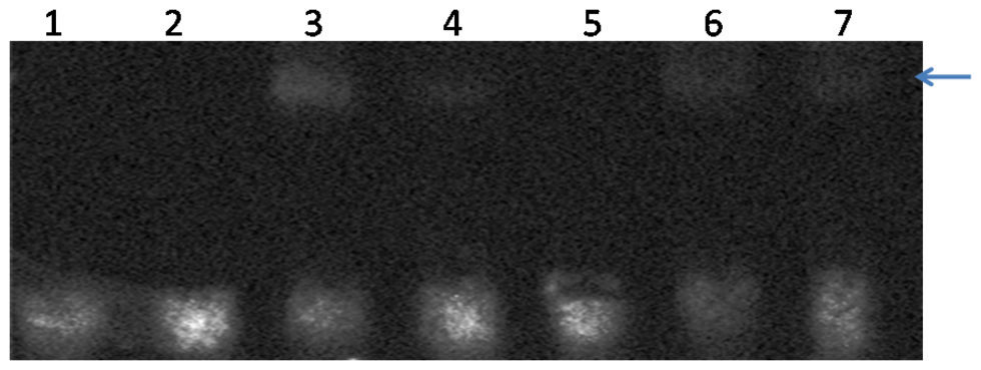
Gel mobility shift assays for box L of the NtPAL promoter. 1, *E.coli*; 2, pGEX-4T-1; 3,4, SbMYB2; 5,6, SbMYB7; 3,5, biotin labeled box L probe; 4,6, biotin labeled and unlabeled box L probes.

Several reports have shown that MYB proteins regulate the expression of *PAL* by combining the box L [24], and box L is present in the *NtPAL* gene [25]. The box L sequence in the promoter sequence of *NtPAL* (GenBank:AB008199) was predicted as ACTTTG using Softberry (linux1.softberry.com). The sequence contains ACTTTG, which has been identified in several gene promoters as a component of binding sites for transcription factor. The interaction between SbMYB2 and SbMYB7 with *NtPAL* promoter sequence was assayed with electrophoretic mobility shift assay (EMSA) experiment. SbMYB2 and SbMYB7 were expressed in *E. coli*, respectively, for the use of EMSA analysis. No binding bands were detected with crude proteins of *E. coli* without or with empty vector (Figure 10 lane 1 and lane 2). SbMYB2 and SbMBY7 specifically bind with the box L sequence, and unlabeled probes inhibit the binding (Figure 10). These results confirmed that SbMYB proteins could combine to the box L sequence of *NtPAL* gene which is the target gene of MYB protein. 

## Discussion

MYB proteins are key factors in regulatory networks controlling development, metabolism and responses to biotic and abiotic stresses. The duplication of R2R3-MYBs plays a key role in generating diversity of gene function [26]. AtMYB75/PAP1, AtMYB90/PAP2, AtMYB113 and AtMYB114 (subgroup 6) control anthocyanin biosynthesis in vegetative tissues [27]. Chalcone synthese (CHS), chalcone isomerase (CHI), flavonol 3-hydroxylase (F3H), flavonol 3’-hydroxylase (F3’H) and flavonol synthase (FLS) are positively regulated by subgroup 7 R2R3-MYBs, and dihydroflavonol-4-reductase (DFR), leucoanthocyanidin dioxygenase (LDOX) and anthocyanidin reductase (ANR) are activated by subgroup 6 R2R3-MYBs [28]. When ectopically expressed in PA-accumulating cells, all R2R3-MYB members in subgroup 4 were able to inhibit PA biosynthesis, suggesting that these proteins have the ability to inhibit the expression of DFR, LDOX and ANR [26]. 

In this study, we isolated nineteen full-length *SbMYB* genes from a *S. baicalensis* cDNA library, and eleven R2R3-MYBs with conserved R2R3 domain were divided into 5 subgroups based on the conservation of the DNA binding domain of *Arabidopsis* MYB proteins. Analysis of new plant genomes suggests that some MYB genes have evolved to fulfill lineage-specific functions [29]. Despite the divergence of the amino-acid sequence outside of the MYB domain, there are some conserved motifs that may contribute to function. Within subgroups that are conserved between divergent species, primary protein structures and biological functions are correlated, such as phenylpropanoid metabolism regulation by R2R3-MYB subgroups 6 [9]. Therefore, protein structure and gene expression patterns can help deduce the functions of new MYB proteins in plants. Using this way, some *Arabidopsis* MYB proteins were predicted to have the function of controlling ﬂavonoids [30] and flavonol [31] biosynthesis. In this study, the transcriptional levels of *SbMYB2* and *SbMYB7* which belong to subgroup 14 were decreased by GA_3_ treatment, whereas the expression of *SbMYB8* which belongs to subgroups 6 was increased after spraying exogenous GA. The transcriptional levels of *SbCHS* and *SbC4H*, two key genes which are involved in baicalein biosynthesis, were also increased. *SbCHS* and *SbC4H* have the similar expression pattern with *SbMYB8*, indicating that SbMYB8 was involved in the flavonoid biosynthesis in *S. baicalensis* based on subgroup classification and co-expressed analysis.

 MYB proteins in subgroup 14 were believed to have functions on plant development [32]. Here, *SbMYB2* and *SbMYB7* which belong to subgroup 14 co-expressed with *SbPALs*, indicating that SbMYB2 and SbMYB7 might be involved in the flavonoid metabolism. To confirm this hypothesis, transgenic tobacco plants overexpressing *SbMYB2* or *SbMYB7* were developed. In transgenic plants, the transcriptional level of some flavonoid biosynthesis-related genes (*NtPAL1*, *NtPAL2*, *NtC4H* and *NtUFGT*) were increased, whereas the transcription levels of *NtCHI* and *NtGT4* were decreased, suggesting that SbMYB2 and SbMYB7 could up-regulate the first step and down-regulate the last step of flavonoid biosynthesis. Consistent with the decreased expression of *NtDH29* and *NtCHI*, the content of dicaffeoylspermidine and quercetin-3,7-O-diglucoside in transgenic tobacco plants was significantly decreased by overexpression of *SbMYB2* or *SbMYB7*, suggesting phenylpropanoid-polyamine conjugates was negatively regulated by above SbMYBs. Dicaffeoylspermidine was a phenylpropanoid-polyamine conjugates and it has been shown that the N-coupling reaction of polyamines to phenolic acids (such as cinnamic, p-coumaric, caffeic, ferulic and sinapic acids) in plants is catalyzed by a speciﬁc class of acyltransferase enzymes. *NaMYB8* silencing induces speciﬁc alterations in the accumulation of coumaroyl-containing metabolites and suppresses caffeoyl- and feruloyl- containing metabolites, and resulted in a strong suppression of dicaffeoylspermidine in Nicotiana [33]. 

Plant hormones affect the accumulation of secondary metabolites and R2R3-type MYB proteins also participate in mediating hormone actions [34]. It has been observed that ABA and GA_3_ treatment decreased *CsPAL* expression level and catechin content [35]. GA_3_ may inhibit the phenylpropanoid pathway through affecting PAL in *Myrica rubra*, pea and carrot [36-38]. Devaiah et al. [39] reported that *AtMYB62* regulated phosphate starvation responses via changes in GA metabolism and signaling. Gibberellin acts through jasmonate to control the expression of *AtMYB21*, *AtMYB24*, and *AtMYB57* to promote stamen filament growth in *Arabidopsis* [40]. Rice GaMYB is an important component of GA signaling in cereal aleurone cells and anther development [41]. In our previous study, the levels of total flavonoids and baicalin and the ratio of baicalin to baicalein in roots of *S. baicalensis* were decreased under water deficit condition after application of GAs, and these decreases were recovered after application of paclobutrazol [23]. The results in this paper also insist that GAs affected flavonoid metabolism in *S. baicalensis*. Over-expression of *Arabidopsis* MYR1 or MYR2 produced GA-deficient symptoms that were rescued by application of GA_3_ [42]. Our result also showed that the transcription level of *NtUFGT* in *SbMYB7-*overexpressing tobacco and *NtHCT* transcripts in *SbMYB2-*overexpressing tobacco was increased. These changes could be rescued by application of GA_3_. These results suggest that *SbMYB2* and *SbMYB7* might regulate the flavonoid biosynthesis through the negative effect on levels of bioactive GA. 

ZmC1 regulates anthocyanin production together with ZmR in maize, suggesting that R2R3 MYB proteins are often involved in the combinatorial interaction of transcription factors for the generation of highly specific expression patterns [43]. The transcriptional levels of *NtPAL1* and *NtPAL2* were increased in transgenic tobacco plants overexpressing *SbMYB2* or *SbMYB7*. *NtPAL2* transcription level was also increased in transgenic plants overexpressing *SbMYB2* and *SbMYB7* after GA_3_ treatment. It has been reported that R2R3-MYB factors regulate the transcriptional activation of *Pinus pinaster PAL* by interaction with the promoter sequence containing AC elements [44], and MYB proteins regulate the expression of *PAL* by combining with the box L [24]. EMSA analysis clearly showed that SbMYB2 and SbMYB7 could combine to the box L sequence of *NtPAL* gene which is the target gene of MYB protein (Figure 10). 

 Within a subgroup, paralogs can control the same metabolic pathway in different cell types as a result of differences in expression patterns [27,30]. AtMYB66/WER and GL1, both clustering together in subgroup 15, can functionally complement each other and display different biological functions only because of their different spatial expression patterns [45]. Although both SbMYB2 and SbMYB7 belong to subgroup 14, they have 62.1% identity at nucleotide acid level and 45.8% identity at amino acid level, and they have different subcellular localization. The transcriptional level of flavonoid biosynthesis-related genes in *SbMYB2*- and *SbMYB7*-overexpressing transgenic tobacco after spray GA_3_ have different expression patterns, indicate that SbMYBs play redundant, but divergent roles in flavonoid biosynthesis and GA response. The results in this study suggested that SbMYB2 and SbMYB7 could affect phenylpropanoid biosynthesis, and SbMYB2 affects flavonoid accumulation through regulating the gibberellin (GA) signaling pathways.

## Materials and Methods

### Plant Materials and Growth Condition

The seeds of *S. baicalensis* were obtained from Institute of Chinese Materia Medica, Academy of Chinese Medical Sciences, Beijing, China), sterilized in 0.5% NaOCl for 5 min, then washed 3 times with sterile water, and placed in petri dishes to germinate. The seedlings 2 weeks after germination were transferred to individual pots (10 seedlings per pot) containing 500 g dried soil in climate chamber at 25°C with 16 h-light photoperiod under well-water condition. GA_3_ (100 uM) were sprayed on leaves of plants one week after transplant of *S. baicalensis* and transgenic tobacco. The leaves were sampled three times at 1, 2 and 3 h after spraying. The sample were rinsed three times in distilled water, and then stored at -80°C for further experiments

### Identification of R2R3-MYB Protein in *S. baicalensis*

To identify R2R3-MYB genes, we performed a BLASTX algorithm [46] at the *S. baicalensis* full-length cDNA library (Yuan et al, unpublished) against the amino acid sequences in NR database (http://www.ncbi.nlm.nih.gov). The functional and structural domains were predicted by InterProScan [47] and Blast2GO [48] analysis, respectively.

### Sequence Analysis

The deduced amino acid sequences were adjusted manually using BioEdit (version 7.0.0) with the default parameters (Pittsburgh Supercomputing Center; http:// www.psc.edu/biomed/genedoc/). Open read frame of 19 R2R3-MYB proteins were performed by BioEdit [49]. Theoretical isoelectric points and molecular weights were predicted using the Compute pI/MW tool on the ExPASy server (http://web.expasy.org/compute_pi/) [50]. The localizations of the deduced proteins were predicted on the ProtComp Version 9.0 (http://linux1.softberry.com/berry.phtml?topic=protcompan&group=programs &subgroup=proloc) as well as SubLoc v1.0 (http://www.bioinfo.tsinghua.edu.cn/SubLoc/). The conserved amino acids were analyzed by protein alignment using such tools as ClustalW and checked manually [51].

### Construction of the Phylogenetic Trees

Phylogenetic analysis of the alignments was performed using ClustalW (Thompson, 1994) and MEGA 4.0 [52] for neighbor-joining analysis. The reliability of these tree topologies was evaluated using bootstrap support with 1000 replicates [53]. The sequences of 126 *Arabidopsis* R2R3-MYB proteins were downloaded from the TAIR *Arabidopsis* genome (http://www.arabidopsis.org/). The predicted proteins of 52 well-known plant R2R3-MYB genes were collected from the National Center for Biotechnology Information (NCBI, http://www.ncbi.nlm.nih.gov/).

### Gene expression analysis in *S. baicalensis*

Total RNA was extracted from plant tissues using Trizol reagent (Invitrogen, USA) and pretreated with RNase-Free DNase (Promega, USA) to eliminate genomic DNA contamination. RNA integrity was analyzed on 1% agarose gel. RNA quantity was determined using a NanoDrop 2000C spectrophotometer (Thermo Scientific, USA).

Semiquantitative RT-PCR was carried out for *SbPAL1* (HM062775), *SbPAL2* (HM062776), *SbPAL3* (HM062777), *SBC4H* (HM062778), *Sb4CL* (HM166767), *SbCHS* (AB008748), *SbUBGAT* (EF512580), *SbGUS* (AB040072), *SbMYBs* and *Sb18S* (FJ527609) using the One-Step RT-PCR kit (TakaRa) with specific primers (Table S7). The *Sb18S* gene was chosen as a loading control. The one-step RT-PCR was done as follows: 94°C for 3 min, 31 cycles of 94°C for 30 s, annealing temperature for 40 s, and 72°C for 40 s, and 72°C for 10 min.

### Subcellular localization

The whole coding sequence of *SbMYB2* and *SbMYB7* was ligated into pE3025 vector [54] digested with *Eco*RI and *Kpn*I to generate plasmids pGEM-SbMYB2 and pGEM-SbMYB7，respectively. In both plasmids, *SbMYB-GFP* fusion genes are under the control of CaMV 35S promoter. The construct was conﬁrmed by sequencing and used for transient transformation of onion (*Allium cepa*) epidermis via a gene gun (Bio-Rad). After 24 h of incubation, GFP ﬂuorescence in transformed onion cells was observed under a confocal microscope (Zeiss, Germany). 

### Transactivation assay

To determine the transactivation activity, the open reading frames of *SbMYB2* and *SbMYB7* were generated by PCR ampliﬁcation, cloned into vector pBD-GAL4 which was digested with *Eco*RI and *Sal*I, to construct pBD-SbMYB2, and pBD-SbMYB7, respectively. The constructs were transformed into YGR2 cells by the lithium acetate-mediated method. The transformed yeast strains were placed on SD/–Trp medium at 28 °C for 2 days. Yeast transformants from SD medium lacking Trp were then transferred and streaked onto solid SD agar lacking Trp/His/Ade (SD/–Trp/–His/–Ade) to score the growth response after 3 days. For the colony-lift ﬁlter assay (X-gal assay), the yeast was transferred to Whatman ﬁlter paper plus X-gal for transcription activation activity analysis within 8 h. pGAL4 and pBD-GAL4 was used as a positive control and negative control, respectively.

### Tobacco transformation

*SbMYB2* and *SbMYB7* fragments were inserted into binary vectors, pCambia1305 to produce p35Spro-SbMYB2 and p35Spro-SbMYB7, respectively. The constructs were then transformed into *Agrobacterium tumefaciens* EHA105. Tobacco (*Nicotiana tabacum*) leaf discs were transformed via an *A. tumefaciens* mediated leaf disc procedure [55] and selected using 50 mg L^-1^ Hygromycin B and 200 mg L^-1^ carbenicillin. After rooting and acclimatization, regenerated plants were grown in a greenhouse to set seeds by self-pollination. T1 transgenic plants were used for further analyses.

### Chemical analysis

To determine flavonoid content, 100 mg powdered tobacco leaf was extracted for 1 h in 1 mL ethyl alcohol. The solution was filtered through a membrane filter (0.2 μm), and flavonoid concentrations were determined using an UPLC-Q-Tof system with a 1.0 mL/min flow rate. UPLC was performed on a diamonsil C18 column (4.6 mm×250 mm, 5 μm). The detection wavelength was set at 354 nm and the column temperature was maintained at 30°C. The mobile phase consisted of acetonitrile-methanoic acid (A; 99.9:0.1, v/v) and deionized water-trifluoroacetic acid (B; 99.9:0.1, v/v). The initial condition was A–B (5:95, v/v) for 20 min, and this was linearly changed to A–B (10:90, v/v) at 20 min, to A–B (20:80, v/v) at 40 min, and to A–B (40:60, v/v) at 60 min. UPLC grade acetonitrile (E. Merck, Darmstadt, Germany) was used for the UPLC analysis. Dicaffeoylspermidine and quercetin-3,7-O-diglucoside were identified using LC-MS and LC-MS/MS. The injection volume of the sample solution was 20 μl, and the experiment was repeated six times.

### Quantitative real-time PCR

Total RNA was reverse-transcribed using Reverse Transcriptase MMLV (Takara, China). PCRs were performed using SYBR Premix Ex Taq kits (TaKaRa, China) following the manufacturer’s instructions and conducted in triplicate using an ABI 7500 Real-Time PCR System (ABI, USA). Gene-specific primers of *NtPAL1* (M84466), *NtPAL2* (D17467), *NtC4H* (AJ937847), *NtCHI* (AB213651), *NtCHS* (AF311783), *NtUFGT* (GQ395697), *NtGT4* (AB176522), *NtAT1* (JN390826), *NtDH29* (JN390824), *NtCCoAMT* (NTU62736), *NtHCT* (NTU62736), *SbMYB2* and *SbYB7* were designed using Primer3 (http://frodo.wi.mit.edu/primer3/). The primer sequences are listed in Table S7. The lengths of PCR products ranged from 100 to 250 bp. *Ntactin* was chosen as an endogenous control in studying gene expressions in various samples of transgenic tobacco. The specificity of amplification was assessed by melting curve analysis, and the relative abundance of genes was determined using the comparative Ct method as suggested in ABI 7500 Software v2.0.1 (ABI).

### Expression of SbMYBs protein in *E.coli*

The open reading frame (ORF) of SbMYB2 and SbMYB7 was cloned into the expression vector pGEX-4T-1 and transformed into Transetta (DE3) chemically competent cells (Beijing TransGen Biotech Co., Ltd, China), respectively. The vector pGEX-4T-1 (+) allows in-frame cloning of PCR products resulting in a GST-tag attached at the N-terminal end of the recombinant protein. Expression of the recombinant protein was induced by adding isopropyl-β-D-1-thiogalactopyranoside (IPTG) and cells were harvested at 9h. 

### Electrophoretic Mobility Shift Assay

The boxes L sequence in the promoter sequence of NtPAL (GenBank:AB008199) was as ACTTTG using Softberry (linux1.softberry.com). Oligonucleotides of boxes L sequence were synthesized and labeled with biotin (Sangon Biotech (Shanghai) Co., Ltd., China) for chemiluminescence using a lightshift chemiluminescent electrophoretic mobility shift assay kit (Pierce). After labeling, complementary labeled strands were mixed together in an equimolar ratio and annealed at room temperature after denaturation at 90°C. Gel mobility shift assays were performed by incubating 0.5 ng of labeled probe with SbMYBs protein and competing oligonucleotides in binding buffer (10 mM Tris-HCl, pH 7.5, 50 mM NaCl, 1 mm dithiothreitol, 1 mm EDTA, 5% glycerol, and 1 μg/μl poly(dIdC) at room temperature for 30 min. Mixtures were size-fractionated on a non-denaturing 46% polyacrylamide gel followed by drying and transfer to nitrocellulose membranes and detection by streptavidin-HRP/chemiluminescence for biotin-labeled probes. 

## Supporting Information

Figure S1 **PCR analysis of transgenic tobacco. M, 2000bp DNA ladder; CK+, gene; CK-, wild-type tobacco.**
(TIF)

Table S1 **Blast results of MYB transcription factors in *Scutellaria baicalensis*.**
(DOC)

Table S2 **Characteristics of R2R3-MYB proteins in *Scutellaria baicalensis*.**
(DOC)

Table S3 **Subcellular localization predicted by ProtComp and SubLoc.**
(DOC)

Table S4 **Real-time RT-PCR analysis of transgenic tobacco.**
(DOC)

Table S5 **Transcriptional level of flavonoid biosynthesis genes in wild-type tobacco.**
(DOC)

Table S6 **Expression patterns of the genes in transgenic tobacco without GA application.**
(DOC)

Table S7 **Primers used in this paper.**
(DOC)
