# Supplementary figures and images for: The Scutellaria baicalensis R2R3-MYB Transcription Factors Modulates Flavonoid Biosynthesis by Regulating GA Metabolism in Transgenic Tobacco Plants
Source: PLoS One. 2013 Oct 15;8(10):e77275. doi: 10.1371/journal.pone.0077275 (PMC3797077; doi:10.1371/journal.pone.0077275)

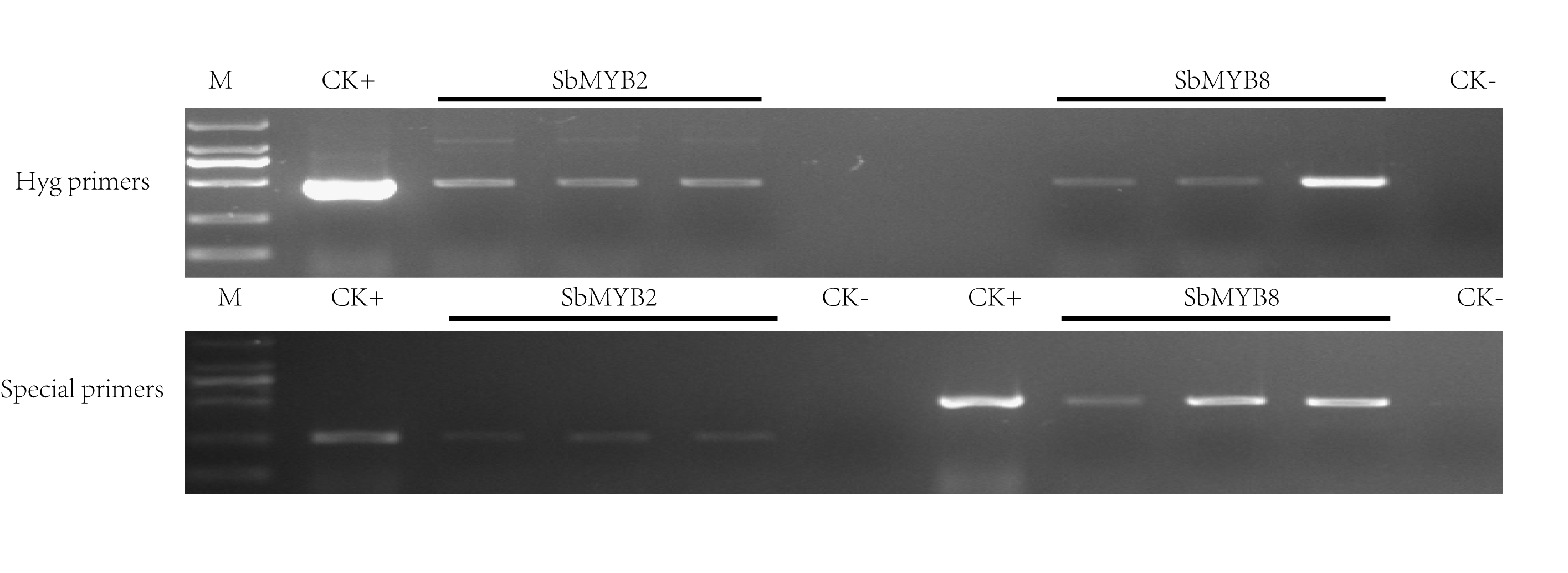

Supplement: Figure S1 — PCR analysis of transgenic tobacco. M, 2000bp DNA ladder; CK+, gene; CK-, wild-type tobacco. (TIF) [file pone.0077275.s001.tif]
