# Supplementary material for: The Scutellaria baicalensis R2R3-MYB Transcription Factors Modulates Flavonoid Biosynthesis by Regulating GA Metabolism in Transgenic Tobacco Plants
Source: PLoS One. 2013 Oct 15;8(10):e77275. doi: 10.1371/journal.pone.0077275 (PMC3797077; doi:10.1371/journal.pone.0077275)
Supplement: Table S1 — Blast results of MYB transcription factors in Scutellaria baicalensis. (DOC) [file pone.0077275.s002.doc]

**Table S1. Blast results of MYB transcription factors in *Scutellaria baicalensis***

| **Gene** | **GenBank No** | **Putative function** | **Species** | **GenBank No.** | **E value** |
| --- | --- | --- | --- | --- | --- |
| SbMYB1 | KF008652 | protein phytoclock 1 | *Arabidopsis thaliana* | AT3G46640 | 2.90e-42 |
| SbMYB2 | KC990835 | transcription factor DcMYB4 | *Daucus carota* | BAF49444 | 4.04E-73 |
| SbMYB3 | KF008653 | MYB transcription factor | *Catharanthus roseus* | ABL63122 | 9.07E-131 |
| SbMYB4 | KF008654 | PREDICTED: similar to MYB transcription factor MYB139 | *Vitis vinifera* | XP_002284400 | 4.77E-73 |
| SbMYB5 | KF008655 | DNA binding protein, putative | *Ricinus communis* | XP_002523835 | 7.84E-67 |
| SbMYB6 | KF008656 | MYB-like DNA-binding protein | *Catharanthus roseus* | CAC19789 | 3.3E-84 |
| SbMYB7 | KC990836 | transcription factor DcMYB4 | *Daucus carota* | BAF49444 | 1.18E-80 |
| SbMYB8 | KF008657 | anthocyanin 2 | *Petunia integrifolia* | ABO21073 | 1.96E-71 |
| SbMYB9 | KF008658 | MYB transcription factor MYB146 | *Glycine max* | ABH02925 | 6.41E-56 |
| SbMYB10 | KF008659 | MYB transcription factor | *Catharanthus roseus* | ABL63122 | 6.42E-79 |
| SbMYB11 | KF008660 | MYB transcription factor MYB161 | *Glycine max* | ABH02906 | 1.28E-61 |
| SbMYB12 | KF008661 | MYB transcription factor 2 | *Vitis pseudoreticulata* | ADC94863 | 1.32E-38 |
| SbMYB13 | KF008662 | R2R3 Myb transcription factor | *Humulus lupulus* | CBI83257 | 3.42E-53 |
| SbMYB14 | KF008663 | DNA binding protein, putative | *Ricinus communis* | XP_002513646 | 3.81E-87 |
| SbMYB15 | KF008664 | myb-related transcription factor | *Solanum lycopersicum* | CAA67600 | 1.27E-95 |
| SbMYB16 | KF008665 | GAMYB | *Triticum monococcum* | AAP40022 | 1.68E-95 |
| SbMYB17 | KF008666 | PREDICTED: similar to MYB transcription factor MYB139 | *Vitis vinifera* | XP_002284400 | 5.07E-62 |
| SbMYB18 | KF008667 | putative Myb-like DNA-binding protein | *Solanum demissum* | AAT40484 | 6.51E-62 |
| SbMYB19 | KF008668 | Myb transcription factor | *Solenostemon scutellarioides* | ABP57085 | 1.61E-74 |
