# Supplementary material for: The Scutellaria baicalensis R2R3-MYB Transcription Factors Modulates Flavonoid Biosynthesis by Regulating GA Metabolism in Transgenic Tobacco Plants
Source: PLoS One. 2013 Oct 15;8(10):e77275. doi: 10.1371/journal.pone.0077275 (PMC3797077; doi:10.1371/journal.pone.0077275)
Supplement: Table S2 — Characteristics of R2R3-MYB proteins in Scutellaria baicalensis. (DOC) [file pone.0077275.s003.doc]

**Table S2. Characteristics of R2R3-MYB proteins in *Scutellaria baicalensis***

| **Gene** | **cDNA**  **length (bp)** | **Protein**  **Length (aa)** | **pI** | **Protein MW (KDa)** | **GOs** | **InterProScan** |
| --- | --- | --- | --- | --- | --- | --- |
| SbMYB2 | 969 | 323 | 7.25 | 36.5 | **P**:regulation of transcription, DNA-dependent; **C**:nucleus; **F**:DNA binding; **F**:transcription factor activity; **P**:response to gibberellin stimulus; **P**:response to abscisic acid stimulus; **P**:blue light signaling pathway; **F**:protein binding; **P**:response to jasmonic acid stimulus; **P**:response to salicylic acid stimulus | PF00249:27-75;  PF00249:82-124 |
| SbMYB5 | 666 | 222 | 6.71 | 27.6 | **P**:response to gibberellin stimulus; **P**:regulation of transcription, DNA-dependent; **P**:response to salicylic acid stimulus; **C**:nucleus; **F**:transcription factor activity; **F**:DNA binding | PF00249:23-67;  PF00249:127-171 |
| SbMYB7 | 909 | 303 | 7.49 | 34.2 | **P**:regulation of transcription, DNA-dependent; **C**:nucleus; **F**:DNA binding; **F**:transcription factor activity | PF00249:14-62;  PF00249:69-111 |
| SbMYB8 | 900 | 300 | 8.58 | 33.4 | - | PF00249:12-59;  PF00249:65-109 |
| SbMYB9* | 651 | 217 | 9.75 | 23.8 | **P**:regulation of transcription, DNA-dependent; **C**:nucleus; **F**:DNA binding; **P**:response to salt stress; **F**:transcription factor activity; **P**:response to gibberellin stimulus; **P**:response to abscisic acid stimulus; **P**:response to auxin stimulus; **P**:response to jasmonic acid stimulus; **P**:response to cadmium ion; **P:**response to ethylene stimulus; **P**:response to salicylic acid stimulus | PF00249:64-108 |
| SbMYB10* | 510 | 170 | 9.38 | 19.1 | **P**:regulation of transcription, DNA-dependent; **C**:nucleus; **F**:DNA binding; **P**:histone H3 acetylation; **F**:transcription factor activity; **P**:response to gibberellin stimulus; **P**:response to abscisic acid stimulus; **P**:response to auxin stimulus; **P**: photoperiodism, flowering; **P**:response to salt stress; **P**:response to jasmonic acid stimulus; **P**:response to cadmium ion; **P**:response to ethylene stimulus; **P**:circadian regulation of gene expression; **P**:response to salicylic acid stimulus; **P:**circadian rhythm | PF00249:58-102 |
| SbMYB11 | 771 | 257 | 6.22 | 28.7 | **P**:regulation of transcription, DNA-dependent; **F**:transcription factor activity; **F**:DNA binding | PF00249:33-81;  PF00249:88-130 |
| SbMYB13 | 705 | 235 | 9.23 | 26.4 | **P**:regulation of transcription, DNA-dependent; **C**:nucleus; **F**:DNA binding; **F**:transcription factor activity; **P**:trichome morphogenesis; **P**:seed germination; **P**:seed coat development; **P**:trichome differentiation; **P**:mucilage biosynthetic process during seed coat development; **P**:regulation of gene expression; **P**:flavonol biosynthetic process; **P**:response to ethylene stimulus; **P**:response to auxin stimulus; **P**:positive regulation of transcription, DNA-dependent; **P**:flavonoid biosynthetic process | PF00249:14-61;  PF00249:67-112 |
| SbMYB14* | 666 | 222 | 9.78 | 24.4 | **P**:regulation of transcription, DNA-dependent; **C**:nucleus; **F**:DNA binding; **F**:transcription factor activity; **P**:response to gibberellin stimulus; **P**:response to abscisic acid stimulus; **P**:response to salt stress; **P**:response to jasmonic acid stimulus; **P**:response to cadmium ion; **P**:response to ethylene stimulus; **P**:response to salicylic acid stimulus; **P**:response to auxin stimulus | PF00249:56-100 |
| SbMYB15* | 774 | 258 | 5.06 | 29.1 | **P**:regulation of transcription, DNA-dependent; **P**:transcription, DNA-dependent; **C**:nucleus; **F**:DNA binding | PF00249:26-73;  PF00249:85-123 |
| SbMYB16* | 732 | 244 | 6.00 | 27.5 | **P**:regulation of transcription, DNA-dependent; **C**:nucleus; **F**:DNA binding; **F**:transcription factor activity; **P**:pollen sperm cell differentiation; **P**:gibberellic acid mediated signaling pathway; **P**:positive regulation of transcription, DNA-dependent; **P**:negative regulation of growth; **P**:response to ethylene stimulus; **P**:response to salicylic acid stimulus; **P**:positive regulation of programmed cell death | PF00249:96-143;  PF00249:149-192 |

P, biological process; F, molecular function; C, cellular component; *, partial sequence
