# Supplementary material for: The Scutellaria baicalensis R2R3-MYB Transcription Factors Modulates Flavonoid Biosynthesis by Regulating GA Metabolism in Transgenic Tobacco Plants
Source: PLoS One. 2013 Oct 15;8(10):e77275. doi: 10.1371/journal.pone.0077275 (PMC3797077; doi:10.1371/journal.pone.0077275)
Supplement: Table S3 — Subcellular localization predicted by ProtComp and SubLoc. (DOC) [file pone.0077275.s004.doc]

**Table S3. Subcellular localization predicted by ProtComp and SubLoc**

| **Name** | **Significant similarity by DBSCAN-P** | **Prediction results** |
| --- | --- | --- |
| SbMYB2 | N | Nuclear/Cytoplasmic |
| SbMYB5 | N | Nuclear/Cytoplasmic |
| SbMYB7 | N | Nuclear/Mitochondrial |
| SbMYB8 | N | Nuclear |
| SbMYB9 | N | Nuclear/Cytoplasmic |
| SbMYB10 | N | Nuclear |
| SbMYB11 | N | Nuclear |
| SbMYB13 | N | Nuclear/Mitochondrial |
| SbMYB14 | N | Chloroplast/Cytoplasmic |
| SbMYB15 | N | Nuclear |
| SbMYB16 | N | Nuclear |
| SbMYB17 | N | Nuclear |
| SbMYB19 | N | Nuclear/Mitochondrial |

N, no signal peptide
