# Supplementary material for: The Scutellaria baicalensis R2R3-MYB Transcription Factors Modulates Flavonoid Biosynthesis by Regulating GA Metabolism in Transgenic Tobacco Plants
Source: PLoS One. 2013 Oct 15;8(10):e77275. doi: 10.1371/journal.pone.0077275 (PMC3797077; doi:10.1371/journal.pone.0077275)
Supplement: Table S4 — Real-time RT-PCR analysis of transgenic tobacco. (DOC) [file pone.0077275.s005.doc]

**Table S4. Real-time RT-PCR of transgenic tobacco**

| **Sample** | **Cт** | **ΔCт** | **2^-ΔCт^** |
| --- | --- | --- | --- |
| **SbMYB2** |  |  |  |
| e10-26-2 | 20.86611 | -6.25979 | 76.63 |
| e10-26-2 | 20.50912 | -6.61678 | 98.14 |
| e10-26-2 | 21.28961 | -5.83629 | 57.13 |
| e10-26-3 | 20.84614 | -5.37707 | 41.56 |
| e10-26-3 | 20.47601 | -5.74720 | 53.71 |
| e10-26-3 | 20.32735 | -5.89586 | 59.54 |
| e10-26-4 | 22.15919 | -6.55125 | 93.78 |
| e10-26-4 | 22.15261 | -6.55783 | 94.21 |
| e10-26-4 | 22.43453 | -6.27591 | 77.49 |
| e10-29-2 | 30.82268 | 3.279736 | 0.10 |
| e10-29-2 | 30.44988 | 2.906943 | 0.13 |
| e10-29-2 | 29.8966 | 2.353655 | 0.20 |
| e10-29-3 | 28.64428 | 3.017076 | 0.12 |
| e10-29-3 | 27.80503 | 2.177829 | 0.22 |
| e10-29-3 | 28.31528 | 2.688081 | 0.16 |
| e10-29-4 | 28.49407 | 0.650206 | 0.64 |
| e10-29-4 | 28.8535 | 1.009642 | 0.50 |
| e10-29-4 | 29.22854 | 1.384682 | 0.38 |
| e10-25-1 | 23.53617 | -4.66525 | 25.37 |
| e10-25-1 | 23.53577 | -4.66565 | 25.38 |
| e10-25-1 | 23.62061 | -4.58081 | 23.93 |
| e10-25-2 | 22.60412 | -3.73338 | 13.30 |
| e10-25-2 | 22.24916 | -4.08834 | 17.01 |
| e10-25-2 | 22.39901 | -3.93849 | 15.33 |
| e10-25-3 | 21.92809 | -4.09034 | 17.03 |
| e10-25-3 | 22.03622 | -3.98221 | 15.80 |
| e10-25-3 | 21.81565 | -4.20278 | 18.41 |
| **SbMYB7** |  |  |  |
| e18-d3 | 30.4963 | 2.687730 | 0.16 |
| e18-d3 | 31.05521 | 3.246640 | 0.11 |
| e18-d3 | 30.85731 | 3.048736 | 0.12 |
| e18-d6 | 30.01163 | 3.390119 | 0.10 |
| e18-d6 | 30.0004 | 3.378892 | 0.10 |
| e18-d6 | 29.82456 | 3.203046 | 0.11 |
| e18-d7 | 25.614 | 2.486170 | 0.18 |
| e18-d7 | 25.85162 | 2.723792 | 0.15 |
| e18-d7 | 25.87564 | 2.747813 | 0.15 |
| e18-53-1 | 28.70185 | -0.18467 | 1.14 |
| e18-53-1 | 28.4014 | -0.48512 | 1.40 |
| e18-53-1 | 28.45233 | -0.43419 | 1.35 |
| e18-53-2 | 27.90802 | 0.220644 | 0.86 |
| e18-53-2 | 27.64664 | -0.04074 | 1.03 |
| e18-53-2 | 27.64006 | -0.04732 | 1.03 |
| e18-53-3 | 27.44571 | -0.22799 | 1.17 |
| e18-53-3 | 27.49568 | -0.17802 | 1.13 |
| e18-53-3 | 27.02312 | -0.65058 | 1.57 |
| e18-b1 | 26.86459 | 1.560234 | 0.34 |
| e18-b1 | 26.40047 | 1.096109 | 0.47 |
| e18-b1 | 26.68541 | 1.381051 | 0.38 |
| e18-b2 | 22.40532 | -4.87538 | 29.35 |
| e18-b2 | 22.52591 | -4.75479 | 27.00 |
| e18-b2 | 22.54786 | -4.73284 | 26.59 |
| e18-b3 | 23.80859 | -3.94904 | 15.44 |
| e18-b3 | 23.78987 | -3.96776 | 15.65 |
| e18-b3 | 23.57041 | -4.18722 | 18.22 |
