# Supplementary material for: The Scutellaria baicalensis R2R3-MYB Transcription Factors Modulates Flavonoid Biosynthesis by Regulating GA Metabolism in Transgenic Tobacco Plants
Source: PLoS One. 2013 Oct 15;8(10):e77275. doi: 10.1371/journal.pone.0077275 (PMC3797077; doi:10.1371/journal.pone.0077275)
Supplement: Table S5 — Transcriptional level of flavonoid biosynthesis genes in wild-type tobacco. (DOC) [file pone.0077275.s006.doc]

**Table S5. Transcriptional level of flavonoid biosynthesis genes in wild-type tobacco.**

| Gene | Ct | | | |
| --- | --- | --- | --- | --- |
|  | 0 h | 1 h | 2 h | 3 h |
| Ntactin | 32.53 | 25.84 | 26.97 | 27.38 |
| NtPAL1 | 32.22 | 25.70 | 26.26 | 24.49 |
| NtPAL2 | 33.77 | 24.45 | 24.96 | 24.02 |
| NtC4H | 31.26 | 27.54 | 27.34 | 26.89 |
| NtCHS | 33.41 | 29.63 | 30.14 | 32.40 |
| NtCHI | 30.39 | 23.39 | 24.49 | 25.80 |
| NtUFGT | 34.83 | 29.41 | 30.83 | 32.57 |
| NtGT4 | 33.73 | 25.55 | 24.75 | 23.93 |
