# Supplementary material for: The Scutellaria baicalensis R2R3-MYB Transcription Factors Modulates Flavonoid Biosynthesis by Regulating GA Metabolism in Transgenic Tobacco Plants
Source: PLoS One. 2013 Oct 15;8(10):e77275. doi: 10.1371/journal.pone.0077275 (PMC3797077; doi:10.1371/journal.pone.0077275)
Supplement: Table S6 — Expression patterns of the genes in transgenic tobacco without GA application. (DOC) [file pone.0077275.s007.doc]

**Table S6. Expression patterns of the genes in transgenic tobacco without GA application**

| Gene | MYB2 | | | | MYB7 | | | |
| --- | --- | --- | --- | --- | --- | --- | --- | --- |
|  | 0 | 1 | 2 | 3 | 0 | 1 | 2 | 3 |
| NtPAL1 | 0.28±0.10 | 0.49±0.39 | 0.37±0.23 | 0.25±0.28 | 0.48±0.55 | 0.22±0.06 | 0.25±0.09 | 0.12±0.11 |
| NtPAL2 | 0.50±0.24 | 0.53±0.52 | 0.37±0.26 | 0.14±0.09 | 0.51±0.56 | 0.25±0.05 | 0.39±0.21 | 0.16±0.13 |
| NtC4H | 0.33±0.14 | 0.32±0.22 | 0.16±0.10 | 0.17±0.18 | 0.15±0.14 | 0.13±0.06 | 0.11±0.03 | 0.11±0.11 |
| NtCHS | 0.37±0.30 | 0.37±0.38 | 0.32±0.39 | 0.12±0.03 | 0.23±0.18 | 0.13±0.09 | 0.28±0.06 | 0.05±0.02 |
| NtCHI | 0.58±0.23 | 0.35±0.47 | 3.11±3.39 | 0.36±0.26 | 0.05±0.04 | 0.43±0.55 | 0.05±0.06 | 0.15±0.08 |
| NtUFGT | 0.32±0.17 | 0.38±0.36 | 0.11±0.03 | 0.10±0.03 | 0.51±0.54 | 0.17±0.09 | 0.29±0.17 | 0.11±0.05 |
| NtAT1 | 0.02±0.01 | 0.18±0.22 | 0.14±0.08 | 0.07±0.09 | 0.02±0.02 | 0.12±0.06 | 0.08±0.04 | 0.05±0.02 |
| NtGT4 | 0.82±0.29 | 0.38±0.41 | 0.74±0.84 | 0.15±0.07 | 0.07±0.05 | 0.10±0.03 | 0.11±0.08 | 0.50±0.74 |
| NtCCoAMT1 | 0.49±0.20 | 0.32±0.37 | 0.42±0.30 | 0.22±0.09 | 0.15±0.16 | 0.13±0.11 | 0.20±0.19 | 0.06±0.02 |
| NtHCT | 0.48±0.17 | 0.37±0.30 | 0.40±0.23 | 0.25±0.16 | 0.16±0.11 | 0.26±0.05 | 0.30±0.17 | 0.25±0.25 |
