# Supplementary material for: The Scutellaria baicalensis R2R3-MYB Transcription Factors Modulates Flavonoid Biosynthesis by Regulating GA Metabolism in Transgenic Tobacco Plants
Source: PLoS One. 2013 Oct 15;8(10):e77275. doi: 10.1371/journal.pone.0077275 (PMC3797077; doi:10.1371/journal.pone.0077275)
Supplement: Table S7 — Primers used in this paper. (DOC) [file pone.0077275.s008.doc]

**Table S7. Primers used** in this paper

| **Primers** | **Sequences (5’ to 3 ’)** | **GenBank No** |
| --- | --- | --- |
| **Semiquantitative RT-PCR** | |  |
| SbPAL1-F | GCGAATAGTGTTCATGATGAGGAT | HM062775 |
| SbPAL1-R | CAATGGCTGCCTTTCCAGTT |  |
| SbPAL2-F | GATTCTGCGTCCAACTCAGTGA | HM062776 |
| SbPAL21-R | GCGTCGGCATTATCCCTG |  |
| SbPAL3-F | GGCCACCAAGATGATCGA | HM062777 |
| SbPAL3-R | CAATGGCCAATCTTGCATTG |  |
| SbC4H-F | GCCGATTCTCTGTATCACTATC | HM062778 |
| SbC4H-R | ATGATTAAAATGATCTTGGCTTT |  |
| Sb4CL-F | ATAATCAAATACAAAGGGTTCCA | AB166767 |
| Sb4CL-R | ACCTGTTTGGATATAAATTGCTT |  |
| SbCHS-F | GCAGTCCACTTATGCTGATTAC | AB008748 |
| SbCHS-R | GTGAAGTTGTCGTTCTCCTTC |  |
| SbUBGAT-F | AGCCAAGGAAGCCATAGTCAAC | EF512580 |
| SbUBGAT-R | CCGAAACAAAGGAAGACGACA |  |
| SbGUS-F | AGAGCAGTGTGAAGATAAGC | AB040072 |
| SbGUS-R | CATAGTAGGTCCAGGCAAG |  |
| Sb18S-F | CGTTGACTACGTCCCTGCCCTT | FJ527609 |
| Sb18S-R | GTTCACCTACGGAAACCTTGTTACGAC |  |
| SbMYB2-F | GTAGTCCAAAAGCACTCACC | KC990835 |
| SbMYB2-R | TCCAAAAAAATAAACTAAGCA |  |
| SbMYB5-F | CTTGGACAGAAGATGAACACAGGT | KF008655 |
| SbMYB5-R | GGGAGATTCGTCGGAGCAG |  |
| SbMYB7-F | ATTACGGTTATGCCCCTGTG | KC990836 |
| SbMYB7-R | GCAATAATCTTGTGGTTTGACTT |  |
| SbMYB8-F | GATGAAAATCCCAAGAGCAACA | KF008657 |
| SbMYB8-R | GCGTCGTCACTTCCACTATCC |  |
| SbMYB12-F | CATTTTACCAGTCCCTCCATC | KF008661 |
| SbMYB12-R | ATAACTTTTCACATCAAGCAACA |  |
| SbMYB13-F | ACCCAAACCCCACAAGGAA | KF008662 |
| SbMYB13-R | ACCGCCCAACAGAAAGTCG |  |
| SbMYB19-F | CCAAGAAGCACAGGAGGACTAAG | KF008667 |
| SbMYB19-R | CTCAAAGAGCGGCGGACA |  |
| **Quantitative real-time PCR** | |  |
| PAL1RTf | TCTCCAAATCTCCTCACAACC | M84466 |
| PAL1RTr | AGCTGCCATTTCCCAATTC |  |
| PAL2RTf | CCAGGATCTTTCTACCCATCC | D17467 |
| PAL2RTr | GTGCAACACCAGCCATTTTT |  |
| NtC4HqPf | GCTGACTAACAACCCTGCTACCTG | AB236952 |
| NtC4HqPr | ATTGCCATTAGCCTCAACGTGCTT |  |
| NtCHIqPf | ACCATTGAGAATTACGTGTTCCCA | AB213651 |
| NtCHIqPr | TCTTCCAAGTAGACGCCGAT |  |
| NtCHSqPf | TAGCCATTTGAAAACCCTAGTAATCGTCCA | AF311783 |
| NtCHSqPr | TACGAAATTCCTCGACGGTCACCA |  |
| NtUFGTqPf | TCCCTTTAGGAAGCCATGCAA C | GQ395697 |
| NtUFGTqPr | TGCCATTTGTTTCTATGACACCA |  |
| NtGT4qPf | TTCAGTTCAACAATTACTCGTGCCAT | AB176522 |
| NtGT4qPr | GAAGGAAGCATGTCAATATTTTCGCAAC |  |
| NtCCoAMT.qPCR.f1 | TTCCCGATGATGGCAAGATTC | NTU62736 |
| NtCCoAMT.qPCR.r1 | GTAATTGTCTTTGTCAGCGTC |  |
| NtHCT.qPCR.f | TGTTCCTGAAACCAGCGTGTCCA | AJ507825 |
| NtHCT.qPCR.f | CCACACATGTCCTGCCAACATCTCG |  |
| NtAT1.qpcr.f | TCACAAGGTTCACTTGTGGCTCTG | JN390826 |
| NtAT1.qpcr.1 | GCATTTGCCTTGAGTTTGCCTAGG |  |
| NtDH29.qPCR.f | ATCAACTAGCCATTAGAATG | JN390824 |
| NtDH29.qPCR.r | CCAAAAATGATTTGCAAGGTC |  |
| NtACT.qF | TCCTGATGGGCAAGTGATTAC | AY594294 |
| NtACT.qR | TTGTATGTGGTCTCGTGGATTC |  |
| SbMYB2.qF2 | GCAGACTCAGATGGCTCAACTACCTCA | KC990835 |
| SbMYB2.qR2 | CCTGGTAATGACCCTGCTATTATGGAC |  |
| SbMYB7.qF2 | CAGGGCAATTACCAGGAAGAACTGACA | KC990836 |
| SbMYB7.qR2 | TACCTTCTTTCACAGGGGCATAACCGTA |  |
